# Evaluation of the Efficacy of a Combined Treatment Using the mTOR-Inhibitor Everolimus and [177Lu]Lu-DOTA-TATE in Nude CD1 Mice with SSTR-Expressing Pancreatic AR42J Xenograft Tumors

**DOI:** 10.3390/biomedicines10123102

**Published:** 2022-12-01

**Authors:** Johannes Zellmer, Hsi-Yu Yen, Lena Kaiser, Franz Josef Gildehaus, Guido Böning, Katja Steiger, Marcus Hacker, Peter Bartenstein, Andrei Todica, Alexander R. Haug, Harun Ilhan

**Affiliations:** 1 Department of Nuclear Medicine, University of Munich, 81377 Munich, Germany; 2 Department of Pathology, Technical University of Munich, 81377 Munich, Germany; 3 German Cancer Consortium (DKTK), German Cancer Research Centre (DKFZ), 69120 Heidelberg, Germany; 4 Comparative Experimental Pathology, Technical University of Munich, 81377 Munich, Germany; 5 Department of Biomedical Imaging and Image-Guided Therapy, Division of Nuclear Medicine, Medical University of Vienna, 1090 Vienna, Austria; 6 Die Radiologie, Private Practice for Radiology, Nuclear Medicine, and Radiation Oncology, 80331 Munich, Germany

**Keywords:** Lu-177-DOTA-TATE, PRRT, everolimus, mTOR inhibitor, neuroendocrine tumors

## Abstract

Therapy options for advanced pancreatic neuroendocrine tumors (pNETs) include the mTOR inhibitor everolimus and peptide receptor radionuclide therapy (PRRT) with [177Lu]Lu-DOTA-TATE, however further optimization in the therapeutic landscape is required as response rates are still low. In this study, we investigated the synergistic and potentially enhanced efficacy of a combined treatment with everolimus and [177Lu]Lu-DOTA-TATE in a mouse model. Baseline [68Ga]Ga-DOTA-TATE PET scans were obtained five days after athymic CD1 mice were inoculated with AR42J tumor cells, before separating the animals into four groups. Group 1 received a placebo, group 2 everolimus, group 3 a placebo and PRRT, and group 4 everolimus and PRRT. The treatment response was monitored by manually measuring the tumor volumes (manual tumor volume, MTV) and conducting sequential [68Ga]Ga-DOTA-TATE PET scans at one, two, and four weeks after treatment induction. The biological tumor volume (BTV) was derived from PET scans using threshold-based volume of interest (VOI) measurements. Tracer uptake was measured semi-quantitatively as a tumor to background ratio (TBR). Mice were euthanized due to excessive tumor growth according to the ethics protocol; blood samples were drawn for the preparation of full blood counts and kidneys were obtained for histological analysis. For the histological assessment, a standardized score (renal damage score, RDS) was used. Full blood counts showed significantly increased numbers of neutrophils and lymphocytes in the groups receiving PRRT. All other parameters did not differ relevantly. In the histological analysis, groups receiving PRRT had a significantly higher RDS, whereas everolimus only tended to cause an increase in the RDS. Mice in groups 1 and 2 had to be euthanized due to excessive tumor growth two weeks after the start of the therapy, whereas follow-up in groups 3 and 4 comprised four weeks. PRRT significantly inhibited tumor growth; the administration of everolimus did not induce an additional effect. A good correlation existed between MTV and BTV. PRRT significantly reduced the TBR. [68Ga]Ga-DOTA-TATE PET is suitable for monitoring tumor growth in the applied model. The high efficacy of [177Lu]Lu-DOTA-TATE is not enhanced by the combination with everolimus.

## 1. Introduction

Neuroendocrine tumors (NETs) represent a rare entity of neoplasms with increasing incidence over the last years and high heterogeneity with respect to the primary tumor site, tumor grading, and pathophysiological properties such as hormonal activity. Despite a relatively high median overall survival of 9.3 years for all patients, there is a wide variety in survival times depending on the primary tumor site and stage. Patients with a primary tumor in the pancreas have an overall survival of 3.6 years, however in the presence of distant metastasis overall survival is only 12 months [1]. Novel treatment strategies established during the last years will hopefully improve these numbers. The RADIANT-3 trial, for instance, showed an improved progression-free survival (PFS) of 11 months for patients suffering from advanced, progressive pancreatic NETs (pNETs) treated with the mTOR inhibitor everolimus compared to 4.6 months for placebo [2]. Furthermore, the NETTER-1 trial significantly changed the therapeutic landscape of NETs. This study was the first phase 3 trial investigating the role of peptide receptor radiotherapy (PRRT) with [177Lu]Lu-DOTA-TATE in patients suffering from NETs, a therapy that has been used in clinical routines on a compassionate use basis for more than 20 years. It showed a significant improvement in PFS as well as quality of life for patients with NETs of midgut origin progressive on first line therapy treated with [177Lu]Lu-DOTA-TATE in combination with a standard dose of octreotide LAR compared to a high dose of octreotide LAR alone [3].

Despite the fact that everolimus and PRRT are used sequentially, a combination of these two therapy options seems reasonable from a theoretical point of view, as everolimus has been proven to enhance the efficacy of (external) radiotherapy in a broad range of solid cancer types in vitro [4,5,6,7,8]. Furthermore, preclinical data suggest that everolimus might even re-sensitize radioresistant tumor endothelial cells [9]. However, due to the proposed synergistic effect and the dissatisfactory results of other combination studies, severe safety concerns are raised. Claringbold et al. reported that the full recommended dose of everolimus was not tolerated in a phase I study combining everolimus and [177Lu]Lu-DOTA-TATE in humans [10].

Bison and Pool treated rats with CA20948 human pancreatic neuroendocrine tumor with everolimus and [177Lu]Lu-DOTA-TATE. They did not find the combined regime to be superior compared to PRRT alone. However, they observed the development of metastases in rats receiving the combined therapy when a complete remission was not achieved [11,12].

In a different rat model, we were recently able to show that a combined treatment with therapeutic doses of both everolimus and [177Lu]Lu-DOTA-TATE does not increase nephro- or hematotoxicity compared to mono-therapies [13]. However, in that previous work, we only evaluated therapy-related toxicity in animals without xenograft tumors.

The current study evaluates the potential synergistic therapeutic effect of everolimus and PRRT with [177Lu]Lu-DOTA-TATE in a mouse model using AR42J pancreatic tumors. Furthermore, an evaluation of the clinically most relevant toxicities, hemato- and nephrotoxicity is performed.

## 2. Materials and Methods

### 2.1. Animals, Tumor Cell Line, and Cultivation and Experimental Design

All animal experiments were performed following institutional guidelines and approved by the ethics committee and Administrative Panel on Laboratory Animal Care (Government of Upper Bavaria, Germany, reference 55.2-1-54-2532-201-12). Seven-week-old female nude CD1 mice weighing 21.5 to 30.6 g (Charles River Laboratories, Sulzfeld, Germany) were used. Mice were fed a standard diet and given free access to water. Body weight was monitored twice weekly.

AR42J cells were cultivated in bovine serum albumin nutritional medium at 37 °C and 5 % CO_2_ atmosphere. These cells overexpress the somatostatin receptor type 2 (SSTR2) and are known to be suitable for [68Ga]Ga-DOTA-TATE PET-imaging [14]. Furthermore, several studies have shown that this cell line seems to be feasible for preclinical PRRT and everolimus trials [15,16].

Mice were inoculated with 5 × 10^6^ tumor cells in the right flank. Five days after the tumor injection, a pre-therapy/baseline [68Ga]Ga-DOTA-TATE PET scan was performed before the mice were randomly divided into four groups. Group 1 received a placebo (n = 7), group 2 everolimus (n = 8), group 3 a placebo in combination with PRRT (n = 7), and group 4 everolimus in combination with PRRT (n = 7). An everolimus + placebo group was omitted, as everolimus is already an established therapy for NET as shown in the RADIANT 3 and 4 trials [2,17]. The dose of everolimus was 5 mg/kg body weight every week and the dose of [177Lu]Lu-DOTA-TATE was 80 MBq once, on the day of the baseline scan. [68Ga]Ga-DOTA-TATE scans were repeated one, two, and four weeks after the baseline scan.

### 2.2. Laboratory Chemical Analysis

A total blood count was performed right before euthanizing the animals at the end of the study. The laboratory analyses were executed according to the manufacturer’s protocols and standardized methods at the Institute of Laboratory Medicine of the Medical Centre of the University of Munich. Blood was not diluted. Blood count analysis was performed using an XN-2000 analyzer (Sysmex, Kobe, Japan).

### 2.3. Pharmaceuticals and Radiopharmaceuticals

Everolimus (formerly known as RAD001) and placebo were kindly provided by Novartis Pharma GmbH (Nuremberg, Germany). We applied a weekly dose of 5 mg/kg body weight as suggested by previously published studies [18]. The pharmaceuticals were freshly prepared from the pre-concentrate once a week right before the oral gavage. Following the manufacturer’s manual, everolimus pre-concentrate was diluted with 5% glucose solution to a concentration of 0.25 mg/mL corresponding to an administered volume of ~0.5 mL. Equivalent amounts of pre-concentrate and glucose solution were used for the preparation of the placebo solution.

No-carrier added 177Lu was obtained from Isotope Technologies Garching GmbH (Garching, Germany). DOTA0, TYR3-octreotate was purchased from ABX advanced biochemical compounds (Dresden, Germany). Radiolabeling was performed according to a previously described protocol [19]. The amount of 80 MBq was chosen according to data by Svensson et al. as a trade-off between moderate toxicity and anti-tumor activity [20]. Radiolabeling of [68Ga]Ga-DOTA-TATE was performed by a radiochemist of the department of nuclear medicine according to protocols described elsewhere labeled with 68Ga obtained from a 68Ge/68Ga generator system (GalliaPharm, Eckert & Ziegler AG, Berlin, Germany) [21]. All radiopharmaceuticals were administered via a tail vein.

### 2.4. PET Imaging and Determination of Tumor Volume

[68Ga]Ga-DOTA-TATE PET imaging was performed with a dedicated small animal PET camera (Inveon Dedicated PET, Preclinical Solutions, Siemens Healthcare Molecular Imaging, Knoxville, TN, USA). After the induction of anesthesia with 1.5% of isoflurane in pure oxygen via a facial mask, 15 MBq of [68Ga]Ga-DOTA-TATE were administered through a tail vein. One static frame was obtained 45 min after the injection of the radiochemical for 30 min. The acquired image was reconstructed using an OSEM 3D algorithm (four iterations) and a MAP 3D algorithm (32 iterations).

In order to analyze the tracer uptake in the tumors, the semi-quantitative measure of the tumor-to-background ratio was calculated by the division of the count rates in standardized volumes of interest (VOIs) which were applied to the tumor and corresponding background regions (M. quadriceps femoris). To determine the tumor VOI, a region of high tracer uptake at the location of the tumor was drawn manually and the voxel of highest activity was selected. This voxel and all neighboring voxels down to a threshold activity of 30 % of the maximum activity were included in the tumor VOI. This method yielded the biological tumor volume (BTV). Tumor volumes were also measured manually determining the maximum diameter of the tumor and two perpendicular diameters using a caliper. The measured tumor volume (MTV) was calculated using the ellipsoid formula V = a × b × c × π/6.

### 2.5. Histopathological Analysis

The histopathological examination was performed on all kidneys of all mice euthanized due to excessive tumor growth. Left and right kidneys were fixed in 4% formaldehyde and stained with HE and PAS. The findings of the histopathological examination were recorded, evaluated, and presented using Excel sheet.

For the evaluation of the kidneys, criteria were used according to the renal damage score system (RDS) described by Rolleman et al. [22]. In case of divergent numbers for the two kidneys of one individual, the mean number was used for further analyses.

### 2.6. Statistical Analysis

Data are expressed as the means of the treatment groups and the corresponding 95%-confidence interval. A *p*-value of *p* < 0.05 was considered statistically significant. Normality and homogeneity of variance were tested using the Shapiro–Wilk test and Levene’s test.

Two-way analysis of variance (ANOVA) was carried out for parameters measured only once in all groups. When normality and/or homogeneity requirements were not met, the Scheirer–Ray–Hare (SRH) test was used, with the administration of everolimus or placebo as one and the treatment with or without [177Lu]Lu-DOTA-TATE as the second factor in both cases. When parameters could only be obtained in two groups, a t-test was used.

The Kruskal–Wallis test was used to test for differences among the ordinally scaled values of the histological grading and Mann–Whitney tests were performed for post-hoc analyses between any two groups applying the Bonferroni correction.

All statistical tests were performed using Microsoft Excel (Microsoft Corporation, Redmond, WA, US) and SPSS Statistics (Version 26, IBM Corporation, Armonk, NY, USA).

## 3. Results

### 3.1. Laboratory Chemical Analysis

The analysis of the total blood count at the end of the trial did not show any significant differences in the erythrocyte, leukocyte or platelet count, the hematocrit, hemoglobin, or the proportion of reticulocytes. Results are displayed in Table 1. A significant increase was only found in the number of neutrophils and lymphocytes due to the PRRT (*p* = 0.003 and *p* = 0.002, respectively). However, the increase in white blood cell count (WBC) due to PRRT was not significant (*p* = 0.051). All other hematologic parameters were also slightly elevated in the groups receiving PRRT. Everolimus increased RBC, hemoglobin, hematocrit, and platelet count and decreased the WBC and the number of neutrophils, monocytes, and lymphocytes, but for none of the parameters was the effect statistically significant.

### 3.2. Histopathological Analysis of the Kidneys

**Figure 1:**
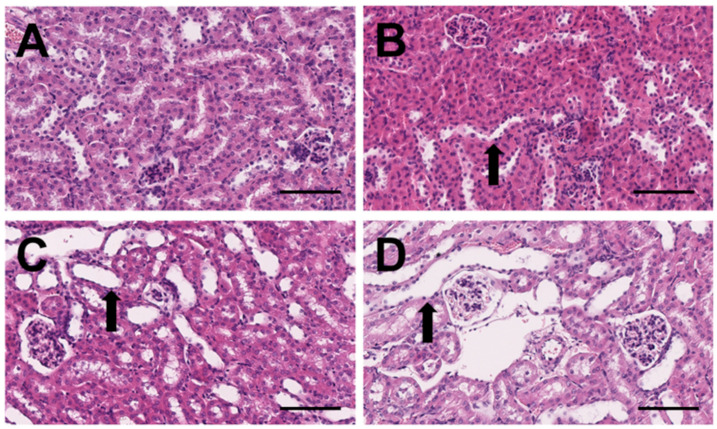
Microscopic images of the kidneys of individual mice in group 1 (**A**), group 2 (**B**), group 3 (**C**), and group 4 (**D**). Tubules are increasingly dilatated in animals of groups 2, 3, and 4 (black arrows). (HE, scale bars: 100 μm).

Examples of the histological sections are presented in Figure 1.

In the glomeruli, no finding was detected in the animals of groups 1 and 2. A minimal to slight multifocal cell reduction and apoptosis were observed in four animals in group 3, and a minimal to slight reduction in five animals in group 4.

In the tubuli, a minimal to slight cell damage or loss of epithelium was observed in five animals in group 2, and a minimal to moderate loss in all animals in groups 3 and 4. A minimal multifocal mononuclear cell infiltration was detected in two animals in group 3. Minimal protein cylinder formation was observed in two animals in group 1 and two animals in group 3, and minimal to slight protein cylinder formation was found in two animals in group 2. A minimal tubulus dilatation was detected in one animal in group 1, and a minimal to slight dilatation in five animals in group 2. In group 3, the tubulus dilatation was found to be minimal to moderate, and in group 4 it was slight to marked in all animals. A minimal multifocal vacuolization in the tubulus epithelium was found in one animal in group 3. A minimal focal regeneration was detected in three animals in group 2.

**Figure 2:**
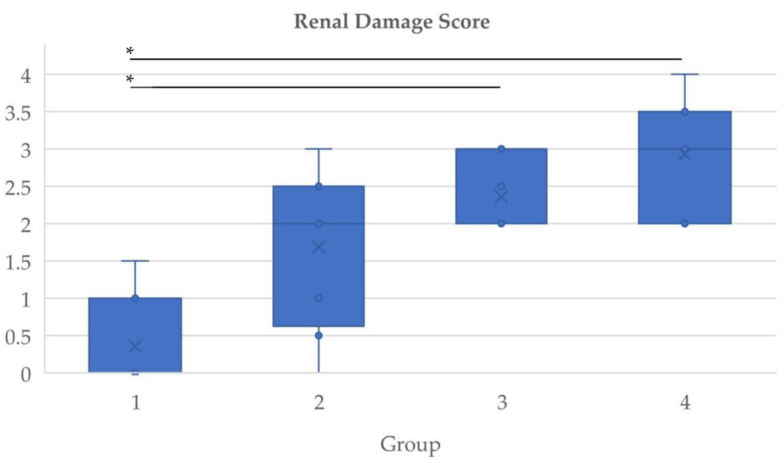
Renal damage scores in the different groups. Circles indicate individual values. Crosses represent means. Boxes cover the interquartile range and whiskers mark minimum and maximum values. Statistically significant differences are marked with an asterisk.

In summary, the median RDS for group 1 is 0 and for groups 2, 3, and 4 it amounts to 2, 2, and 3, respectively. The distribution of the scores is depicted in Figure 2.

The Kruskal–Wallis test showed significant differences in the RDS values (*p* = 0.001) and the post-hoc analyses revealed a significantly lower RDS in the placebo group compared to groups receiving PRRT (*p* = 0.007 for group 3 and *p* = 0.008 for group 4). No significant difference was found between the everolimus and the PRRT group. Combined treatment induced a higher RDS compared to everolimus monotherapy without being statistically significant (*p* = 0.22).

### 3.3. Tumor Growth

Mice were euthanized due to the penetration of the tumor through the skin or the tumor size, following animal welfare regulations as described in the ethics approval. Mice receiving placebo (group 1) had to be euthanized on day 15 (n = 3) or day 19 (n = 4). Mice receiving everolimus (group 2) also had to be euthanized on day 15 (n = 4) and day 19 (n = 4). Mice receiving [177Lu]Lu-DOTA-TATE and a placebo or everolimus (groups 3 and 4) were euthanized on day 33. One mouse in group 4 was lost on day 26 due to aspiration during the gavage of everolimus.

**Figure 3:**
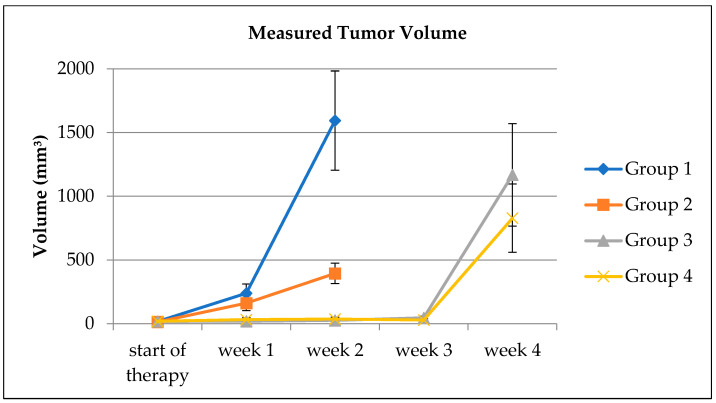
Time course of the manually measured tumor volumes averaged in the different groups. Error bars indicate the standard error of the mean.

Tumor volumes were distributed homogeneously at the start of the treatment. Figure 3 shows the progression in tumor volume in the different groups.

At day 19, two weeks after the start of the treatment, MTVs were 1.6 ± 1.3 cm^3^ in group 1, 0.39 ± 0.26 cm^3^ in group 2, 0.026 ± 0.037 cm^3^ in group 3, and 0.036 ± 0.034 cm^3^ in group 4. Since the Shapiro–Wilk test revealed a significant deviation from normality, an SRH test was performed to analyze the differences between groups. Results showed significantly smaller MTVs only for [177Lu]Lu-DOTA-TATE (*p* < 0.001) but not for everolimus (*p* = 0.55).

MTVs did not differ significantly between groups 3 and 4 at day 33 (*p* = 0.497).

At euthanasia, the averaged masses of the xenografts were 1.2 ± 0.7 g in group 1, 0.8 ± 0.7 g in group 2 and did not differ significantly (*p* = 0.363). In group 3, the mean tumor mass was 1.1 ± 1.1 g, and in group 4 it was 0.7 ± 0.8 g. Again, no significant difference was found (*p* = 0.481).

### 3.4. Biological Tumor Volume

**Figure 4:**
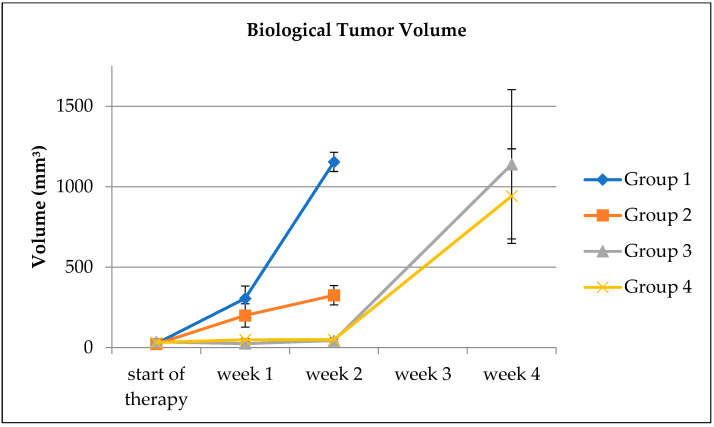
Time course of the tumor volumes determined from the PET scans averaged in the different groups. Error bars indicate the standard error of the mean.

**Figure 5:**
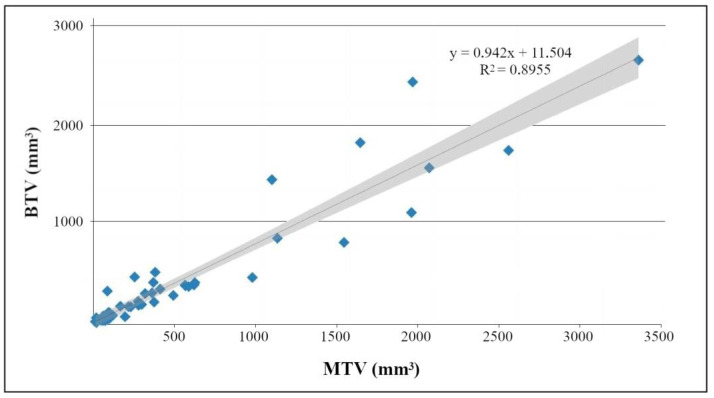
Correlation of manually measured tumor volumes (MTV) and the tumor volumes obtained from the PET scans (BTV). The gray area marks the confidence band of the linear regression.

The BTVs obtained using the thresholding method (Figure 4) were plotted versus the respective MTVs (Figure 5). The result of the linear regression was BTV = 0.942 × MTV + 0.012 cm^3^ (95%-CI for the correlation coefficient [0.9010, 0.983]) with the determination coefficient R^2^ = 0.8955.

### 3.5. Tumor to Background Ratio

**Figure 6:**
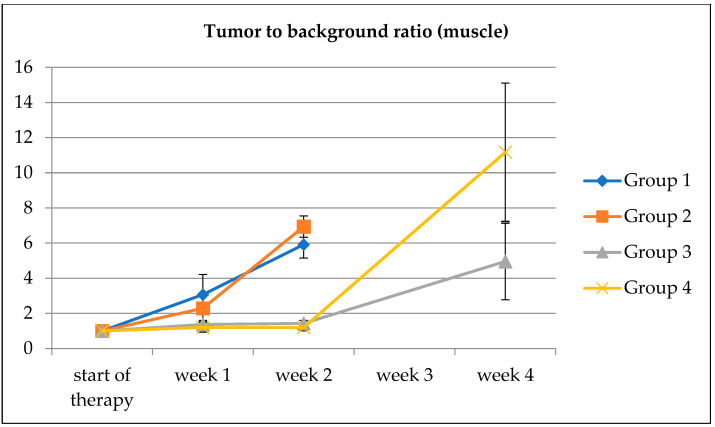
Tumor to background ratios with VOIs in the muscle (M. quadriceps femoris) as background. Error bars indicate the standard error of the mean.

TBR was determined separately for each scan and normalized to the individual TBRs in the baseline scans. TBRs are plotted in Figure 6 with the muscle as the background region. SRH test showed a significantly lower TBR for PRRT (*p* < 0.001) but not for everolimus (*p* = 0.98) as a factor.

## 4. Discussion

In the NCCN guidelines for the management of neuroendocrine and adrenal tumors, PRRT and everolimus represent first- or second-line therapy options in patients with metastatic NETs [23]. However, as objective response rates are relatively low, further optimization of therapy algorithms and sequences is needed. Our approach in the current preclinical trial was to combine both therapeutic options in AR42J tumor-bearing nude mice.

The results regarding hematotoxicity are in line with our findings in rats as previously reported [13]. Everolimus induced an increase in hemoglobin concentration and a decrease in white blood count. These findings did not reach statistical significance. However, the (statistically significant) increase in neutrophil, as well as the lymphocyte count due to PRRT, was not observed in the previous study. This divergence might be caused by the substantially shorter interval between the application of PRRT and the blood analysis and is considered a temporary, potentially reversible effect. There was no essential deterioration of the blood count in the group receiving the combined therapy compared to PRRT and placebo. In summary, with the given methods, no severe high-grade toxicity in terms of blood parameter elevation was observed with the treatment dose chosen for this study.

Histological evaluation revealed significant nephrotoxicity in mice receiving PRRT regardless of the addition of everolimus when compared to the group receiving only placebo. Considering the dose threshold of ~ 60 MBq [177Lu]Lu-DOTA-TATE for nephrotoxicity described by Svensson et al. [20], these findings are not surprising. The main consideration behind escalating PRRT doses was to maximize potential therapeutic synergistic effects by adding everolimus. In this regard, the combined treatment did not show increased nephrotoxicity compared to PRRT alone. Thus, we conclude that nephrotoxicity is also acceptable in the combined regime as implemented in this trial and is mainly dependent on the PRRT dose. As nephrotoxicity is rare when using [177Lu]Lu-DOTA-TATE at standard doses of 7.4 GBq per administration, these results might not be transferable to human data anyway.

A significant deceleration in tumor growth was found for the treatment with [177Lu]Lu-DOTA-TATE. The observed delay in tumor growth due to PRRT is similar to the results reported by Cullinane et al., who evaluated the effect of [177Lu]Lu-DOTA-TATE in a combined regime with the PARP-inhibitor talazoparib using Balb/c nude mice with AR42J-tumors [16]. In this study, mice treated with [177Lu]Lu-DOTA-TATE showed tumor regression for two weeks after treatment and an overall survival of 37 days. This fits our observation of a two-week longer observation period after PRRT and the sacrifice due to tumor growth on day 28. The tendentially poorer performance of the mice used in our trial could be explained by the additional stress due to the PET scans and the necessary anesthesia.

Experimental data have demonstrated the role of the mTOR-signaling pathway in AR42J cells. mTOR-inhibition blocks signaling from mitogenic growth factors and mTOR-activation protects against inflammation [24,25]. Therefore, the treatment with everolimus should not only inhibit tumor growth but also aggravate the inflammatory response to DNA damage caused by PRRT. However, the current paper will not allow detailed insight into tumor biology and response pathways as it mainly evaluates the response assessment using SSTR-PET and tumor growth.

[68Ga]Ga-DOTA-TATE PET scans represent a suitable method for in vivo determination and monitoring of tumor burden with a good correlation of PET-derived BTVs and manually measured MTVs.

Despite being not significant, the addition of everolimus increased the TBR, which fits the clinical observation that everolimus can induce somatostatin receptor expression [26]. However, no significant differences in tumor size were observed.

The efficacy of everolimus as a radiosensitizer has been shown for external beam radiotherapy as mentioned earlier [4,5,6,7,8]. Unlike external beam radiotherapy, however, PRRT applies heterogeneous and prolonged irradiation and relatively low dose rates. Whether these differences urge the need for the development of distinctive radiosensitizers for PRRT is currently the subject of discussion as the underlying radiobiology is not yet fully understood [27]. Nonetheless, our preclinical data and a phase I trial in patients indicate that the combination of PRRT and everolimus might be associated with higher toxicity without higher anti-tumoral effects [10,13].

Unlike Bison, Pool, et al. who treated CD20948 bearing rats with a combination of everolimus and [177Lu]Lu-DOTA-TATE [11,12], we could not observe the development of metastasis. This divergence may be attributed to our rather short observation period, as mice had to be sacrificed according to the underlying study protocol as approved by the animal welfare committee that could be overcome in further experiments by resecting the primary tumor. Furthermore, our study represents localized disease, which is not the case in the patient scenario when PRRT and/or everolimus is applied as most patients suffer from metastatic disease.

In summary, the combination of PRRT and everolimus in the treatment of neuroendocrine tumors remains complex even in a preclinical setting. In an early Phase 1 clinical trial the combined regime could only be administered with reduced doses of everolimus due to unacceptable toxicity, further questioning this approach [10]. However, research into alternative options to optimize PRRT for neuroendocrine neoplasms like precise dosimetry for dose escalation seems promising.

## 5. Conclusions

[68Ga]Ga-DOTA-TATE PET scans are a suitable method for monitoring tumor size in SSTR2-positive AR42J tumors in mice. Combined treatment with everolimus and [177Lu]Lu-DOTA-TATE does not induce a significantly increased toxicity in this model. PRRT with [177Lu]Lu-DOTA-TATE shows good anti-tumor activity in this model independent of a combination with everolimus without further synergistic effects for the combined treatment.

## Figures and Tables

**Table 1:** Results of the total blood count performed at euthanasia. PRRT causes a significant increase in lymphocyte and neutrophil counts. *p*-values result from a two-way ANOVA of the four groups.

	Placebo	Everolimus	Placebo + [177Lu]Lu-DOTA-TATE	Everolimus + [177Lu]Lu-DOTA-TATE	*p*-Values
RBC (10^12^/L)	6.15 ± 1.32	7.96 ± 1.26	8.01 ± 3.57	7.82 ± 2.17	0.154
Hemoglobin (g/L)	99 ± 18	126 ± 18	114 ± 54	124 ± 31	0.181
Hematocrit	0.331 ± 0.058	0.419 ± 0.056	0.413 ± 0.103	0.414 ± 0.088	0.080
Reticulocytes (‰)	60.4 ± 30.5	39.6 ± 9.8	81.0 ± 88.9	71.7 ± 49.9	0.252
Platelets (10^9^/L)	729 ± 459	965 ± 374	1028 ± 743	1079 ± 200	0.469
WBC (10^9^/L)	3.29 ± 2.07	2.58 ± 1.03	5.06 ± 2.59	3.78 ± 1.80	0.158
Neutrophils (10^9^/L)	0.83 ± 0.34	0.97 ± 0.49	1.91 ± 0.63	1.26 ± 0.58	0.013 *
Monocytes(10^9^/L)	0.09 ± 0.06	0.05 ± 0.03	0.11 ± 0.07	0.10 ± 0.09	0.306
Lymphocytes (10^9^/L)	0.94 ± 0.88	0.76 ± 0.34	2.36 ± 1.79	1.89 ± 1.06	0.014 *

Statistically significant differences are marked with an asterisk.

## Data Availability

The datasets generated during and/or analyzed during the current study are available from the corresponding author on reasonable request.
